# Variants in the *ASB10* Gene Are Associated with Primary Open Angle Glaucoma

**DOI:** 10.1371/journal.pone.0145005

**Published:** 2015-12-29

**Authors:** Shazia Micheal, Humaira Ayub, Farrah Islam, Sorath Noorani Siddiqui, Wajid Ali Khan, Farah Akhtar, Raheel Qamar, Muhammad Imran Khan, Anneke I. den Hollander

**Affiliations:** 1 Department of Ophthalmology, Radboud University Nijmegen Medical Centre, Nijmegen, the Netherlands; 2 Department of Biosciences, COMSATS Institute of Information Technology, Islamabad, Pakistan; 3 Al-Shifa Eye Trust Hospital Jhelum Road, Rawalpindi, 46000, Pakistan; 4 Al-Nafees Medical College & Hospital, Isra University, Islamabad, 45600, Pakistan; 5 Department of Human Genetics, Radboud University Nijmegen Medical Centre, Nijmegen, the Netherlands; Casey Eye Institute, UNITED STATES

## Abstract

**Background:**

Recently nonsynonymous coding variants in the ankyrin repeats and suppressor of cytokine signaling box-containing protein 10 (*ASB10*) gene were found to be associated with primary open angle glaucoma (POAG) in cohorts from Oregon and Germany, but this finding was not confirmed in an independent cohort from Iowa. The aim of the current study was to assess the role of *ASB10* gene variants in Pakistani glaucoma patients.

**Methods:**

Sanger sequencing of the coding exons and splice junctions of the *ASB10* gene was performed in 30 probands of multiplex POAG families, 208 sporadic POAG patients and 151 healthy controls from Pakistan. Genotypic associations of individual variants with POAG were analyzed with the Fisher’s exact or Chi-square test.

**Results:**

In total 24 variants were identified in POAG probands and sporadic patients, including 11 novel variants and 13 known variants. 13 of the variants were nonsynonymous, 6 were synonymous, and 5 were intronic. Three nonsynonymous variants (p.Arg49Cys, p.Arg237Gly, p.Arg453Cys) identified in the probands were not segregating in the respective families. This is not surprising since glaucoma is a multifactorial disease, and multiple factors are likely to be involved in the disease manifestation in these families. However a nonsynonymous variant, p.Arg453Cys (rs3800791), was found in 6 sporadic POAG patients but not in controls, suggesting that it infers increased risk for the disease. In addition, one synonymous variant was found to be associated with sporadic POAG: p.Ala290Ala and the association of the variant with POAG remained significant after correction for multiple testing (uncorrected p-value 0.002, corrected p-value 0.047). The cumulative burden of rare, nonsynonymous variants was significantly higher in sporadic POAG patients compared to control individuals (p-value 0.000006).

**Conclusions:**

Variants in *ASB10* were found to be significantly associated with sporadic POAG in the Pakistani population. This supports previous findings that sequence variants in the *ASB10* gene may act as a risk factor for glaucoma.

## Introduction

Glaucoma is a group of heterogeneous optic neuropathies and a leading cause of irreversible blindness, affecting approximately 70 million individuals worldwide [1, 2]. One of the major risk factors for glaucoma is an abnormal elevation of intraocular pressure (IOP). Glaucoma causes an irreversible destruction of the optic nerve, which ultimately affects the central visual pathway. This involves the degeneration and death of the retinal ganglion cells, which leads to a progressive deterioration of the visual field [3].

The cellular and molecular mechanisms underlying glaucoma are not yet fully understood. Family-based linkage analysis and case-control studies have shown that genetic variants contribute to the pathogenesis of POAG [4–8]. At least 16 chromosomal loci for POAG have been reported [4–6]. To date, several causative genes and 16 POAG-associated loci have been identified, including myocilin (*MYOC*/GLC1A; MIM 601652) [7, 8], optineurin (*OPTN*/GLC1E; MIM 137760) [9], WD repeat domain 36 (*WDR36*/GLC1G; MIM 609669) [10, 6], cytochrome P450 1B1 (*CYP1B1*; MIM 231300) [11,12]. In addition, genome-wide association studies have detected several POAG-associated gene variants, some potentially acting at the level of the trabecular meshwork (associated with elevated IOP), and others possibly at the level of the retinal ganglion cells [13–16].

Recently, ankyrin repeats and suppressor of cytokine signalling (SOCS) box-containing protein 10 (*ASB10*) has been identified as a novel POAG candidate gene (MIM 603383) located at chromosome 7q35–q36, the *GLC1F* locus. Further support was provided by the additional screening of the POAG patients from Oregon and Germany [17], but this was not confirmed in an independent POAG cohort from Iowa [18]. In the current study we further investigated the role of the *ASB10* gene in glaucoma families and sporadic POAG patients from Pakistan.

## Materials and Methods

### Subjects

Thirty families with juvenile- and adult-onset POAG (age range 16–60 years), 208 sporadic Pakistani POAG patients with a mean age of 55.3±1.2 years (51% male and 49% female), and 151 healthy controls with a mean age of 53.7±1.1 years (53% male, 47% female) were included in this study. Probands of all the families included in the current study were excluded for the variants in the *CYP1B1*, *MYOC*, *OPTN*, *WDR36* genes.

### Ethics statement

The study adhered to the guidelines of the Declaration of Helsinki and was approved by the Institutional Review Board of Al-Shifa Eye Trust Hospital. After signed informed consents were obtained, blood samples were collected for DNA isolation.

### Clinical evaluations

Complete ophthalmic examinations were performed for patients and controls. Juvenile-onset POAG was diagnosed between age 3 years and early adulthood (<35 years). Adult-onset POAG was diagnosed at adulthood, usually after 35 years of age. Briefly, for POAG patients the inclusion criteria were high IOP (>21 mmHg) measured using Goldmann applanation tonometry, a cup-to-disc ratio (CDR) >0.7 with thinning or notching of the disc rim [19], and visual field defects. Visual field defects typical of glaucoma such as arcuate scotoma, nasal step and paracentral scotoma were determined with a Humphrey Field Analyzer (Zeiss Humphrey Systems, Dublin, CA, USA), and an open anterior chamber angle was confirmed with gonioscopy. Although high IOP is not part of the definition of glaucoma, we used strict criteria to exclude other types of glaucoma in our cohort. Controls and cases were matched for age, gender and ethnicity. The same detailed ophthalmological examinations were done for the controls as for the glaucoma patients, and only control individuals without glaucoma were selected.

### DNA isolation and genetic analysis

A conventional phenol-chloroform method was used for the extraction of genomic DNA from whole blood [20]. Primers flanking the entire coding sequence of *ASB10* were designed with Primer3 software (primer sequences and PCR conditions are available on request). The amplified region covered at least 50 base pairs into each intron to screen for potential mutations affecting splicing. PCR products were visualized on 2% agarose gel and purified with PCR clean-up purification plates (NucleoFast^®^ 96 PCR, MACHEREY-NAGEL, Germany), according to the manufacturer’s protocol. Purified PCR products were analyzed by Sanger sequencing in an automated DNA sequencer (Big Dye Terminator, version 3 on a 3730 DNA analyzer; Applied Biosystems, Inc., Foster City, CA). Sequencing results were aligned with the reference sequence (obtained from the hg19 human genome build) and analyzed using Vector NTI Advance (TM) 2011 software (Invitrogen).

### *In silico* analysis

In addition, the pathogenicity of *ASB10* missense variants was evaluated by publically available tools including PhyloP, Grantham, PolyPhen2, and SIFT [21]. Evolutionary conservation was determined for variants which were predicted pathogenic by PolyPhen2 and SIFT, or had a high Grantham distance or PhlyoP score. To assess the amino acid conservation, orthologous ASB10 protein sequences of various species were aligned using Vector NTI Advance (TM) 2011 software. The amino acid sequences were obtained from protein sequence database UniProt (http://www.uniprot.org).

### Statistical analysis

The genotype frequency of individual sequence variants in the *ASB10* gene was compared between sporadic POAG patients and control individuals using a Fisher’s exact test for rare variants (n<5), and a Chi-square test for common variants. P-value ≤0.002 was considered significant after correction for multiple testing (α/number of variants) [22], (https://www.easycalculation.com/statistics/bonferroni-correction-calculator.php).

## Results

Analysis of the *ASB10* gene in 30 probands (one per family) of Pakistani glaucoma families revealed six nonsynonymous, two synonymous, and two intronic variants (Tables 1 and 2).

**Table 1:** Non-synonymous variants identified in the *ASB10* gene in glaucoma patients.

Nucleotide change	Amino acid change ±±	Previously labelled±	rs-number	Control (n = 151)	*Proband (n = 30)	Sporadic Patient (n = 208)	P-value Un-corrected [corrected]	PhyloP	Grantham dist	SIFT Score	Poly Phen±	EVS MAF
c.145C>T	p.Arg49Cys	c.272-327C>T	rs142736544	0	CT = 1 (3.3%)	CT = 1 (0.4%)	NS	1.17	180	D	1.00	0.00007
c.184G>A	p.Val62Met	c.272-288G>A	Novel	0	0	GA = 2 (0.9%)	NS	0.61	21	D	0.82	Absent
c.687G>T	p.Glu229Asp	p.Glu214Asp	Novel	GT = 1 (0.6%)	0	GT = 1 (0.4%)	NS	-0.12	45	T	-0.12	Absent
c.709C>G	p.Arg237Gly	p.Arg222Gly	rs61735708	0	CG = 2 (6.6%)	CG = 1 (0.4%)	NS	1.66	125	T	0.63	0.005
c.885G>T	p.Gln295His	p.Gln280His	Novel	0	0	GT = 1 (0.4%)	NS	0.12	24	T	0.002	Absent
c.887G>A	p.Arg296Gln	p.Arg281Gln	Novel	0	0	GA = 4 (1.9%)	NS	1.01	43	T	1.00	Absent
c.910C>T	p.Arg304Cys	p.Arg289Cys	rs61735130	0	0	1 (0.4%)	NS	3.11	180	D	0.99	0.002
c.1025T>C	p.Leu342Pro	p.Leu327Pro	Novel	1 (0.6%)	0	1 (0.4%)	NS	0.77	98	D	0.77	Absent
c.1075G>A	p.Val359Ile	p.Val344Ile	Novel	GG = 137 (90.7) GA = 13(8.6%) A = 1(0.6%)	GG = 27 (90%) GA = 3(10%)	GG = 200 (96%) GA = 5(2.4%) AA = 3(1.4%)	0.02 [0.38]	1.09	29	T	0.98	0.0003
c.1114C>T	p.Arg372Cys	p.Arg357Cys	rs62489646	CT = 4 (2.6%)	2 (6.6%)	9 (4.3%)	NS	1.25	180	D	1.00	Absent
c.1204C>A	p.Pro402Thr	p.Pro387Thr	rs919533	CA = 18 (11.9%)	CA = 4 (13.3%)	CA = 15 (7.2%)	NS	1.09	38	T	0.62	Absent
c.1340G>A	p.Arg447His	p.Arg432His	Novel	0	0	1 (0.4%)	NS	0.77	29	D	0.77	Absent
c.1357C>T	p.Arg453Cys	p.Arg438Cys	rs3800791	0	1 (3.3%)	6 (2.8%)	0.04 [0.62]	-0.12	180	T	0.005	0.009

*Proband column includes only one proband per family. P-values were given only for controls versus sporadic patients,

NS; not significant,

D; Deleterious:

T; Tolerated.

^±±^ASB10 reference sequence is NM_001142459.1 and NP_001135931.2 (isoform 1) determined.

^±^ ASB10 reference sequence is NM_080871.3 and NP_543147.2 (isoform 3).

Variant with PolyPhen score >0.5 is considered to be probably damaging. Variants with PhyloP score >2 or Grantham score >80 are considered to be pathogenic.

EVS MAF: Exome variant server Minor allele frequency.

**Table 2:** Synonymous and intronic variants identified in the *ASB10* gene in glaucoma patients

Location	Nucleotide change	A.Acid change ±±	Previously labelled ±	Mutation Status	Control (n = 151)	*Proband (n = 30)	Sporadic Patient (n = 208)	P-value uncorrected [corrected]	EVS MAF
Exon 2	c.270C>T	p. = (p.Ser90Ser)	c.272-202C>T	rs146732530	0	0	1 (0.4%)	NS	0.0002
Intron 2	c.316+10G>A	Intronic	c.272-146G>A	rs10275136	10 (6.6%)	2 (6.6%)	7 (3.3%)	NS	0.120
Intron 2	c.316+9C>T	Intronic	c.272-147C>T	Novel	6 (3.9%)	0	2 (0.9%)	NS	Absent
Intron 2	c.317-75C>T	Intronic	c.272-75C>T	rs10275219	0	0	1 (0.4%)	NS	Absent
Exon 2	c.129G>A	p. = (p.Pro43Pro)	c.272-343G>A	rs144038078	0	0	2 (0.9%)	NS	0.001
Exon 4	c.738C>T	p. = (p.Ala246Ala)	p. = (p.Ala231Ala)	Novel	0	0	2 (0.9%)	NS	0.00007
Exon 4	c.798C>T	p. = (p.Ala266Ala)	p. = (p.Ala251Ala)	rs61743170	CT = 17 (11.2%)	1 (3.3%)	15 (7.2%)	NS	0.086
Exon 4	c.855A>G	p. = (p.Ala285Ala)	p. = (p.Ala270Ala)	Novel	0	0	1 (0.4%)	NS	Absent
Exon 4	c.870G>C	p. = (p.Ala290Ala)	p. = (p.Ala275Ala)	rs2253592	GG = 83 (55.0%) GC = 50 (33.1%) CC = 18 (11.9%)	GG = 12 (40%) GC = 15 (50%) CC = 3 (10%)	GG = 96 (46.2%) GC = 102 (49%) CC = 10 (4.8%)	0.002 [0.047]	0.93
Intron 4	c.1104+69G>A	Intronic	c.1059+69G>A	Novel	1 (3.3%)	0	1 (0.2%)	NS	Absent
Intron 5	c.1218+42T>C	Intronic	c.1173+42T>C	rs310598	TT = 81 (53.6%) TC = 50 (33.1%) CC = 20 (13.3%)	TT = 14 (46.7%) TC = 13 (43.3%) CC = 3 (10%)	TT = 123 (59.1%) C = 73 (35.1%) CC = 12 (5.8%)	0.048 [0.62]	Absent

*Proband column includes only one proband per family. P-values were given only for controls versus sporadic patients,

±±ASB10 reference sequence is NM_001142459.1 and NP_001135931.2 (isoform 1) determined.

± ASB10 reference sequence is NM_080871.3 and NP_543147.2 (isoform 3).

**Fig 1:**
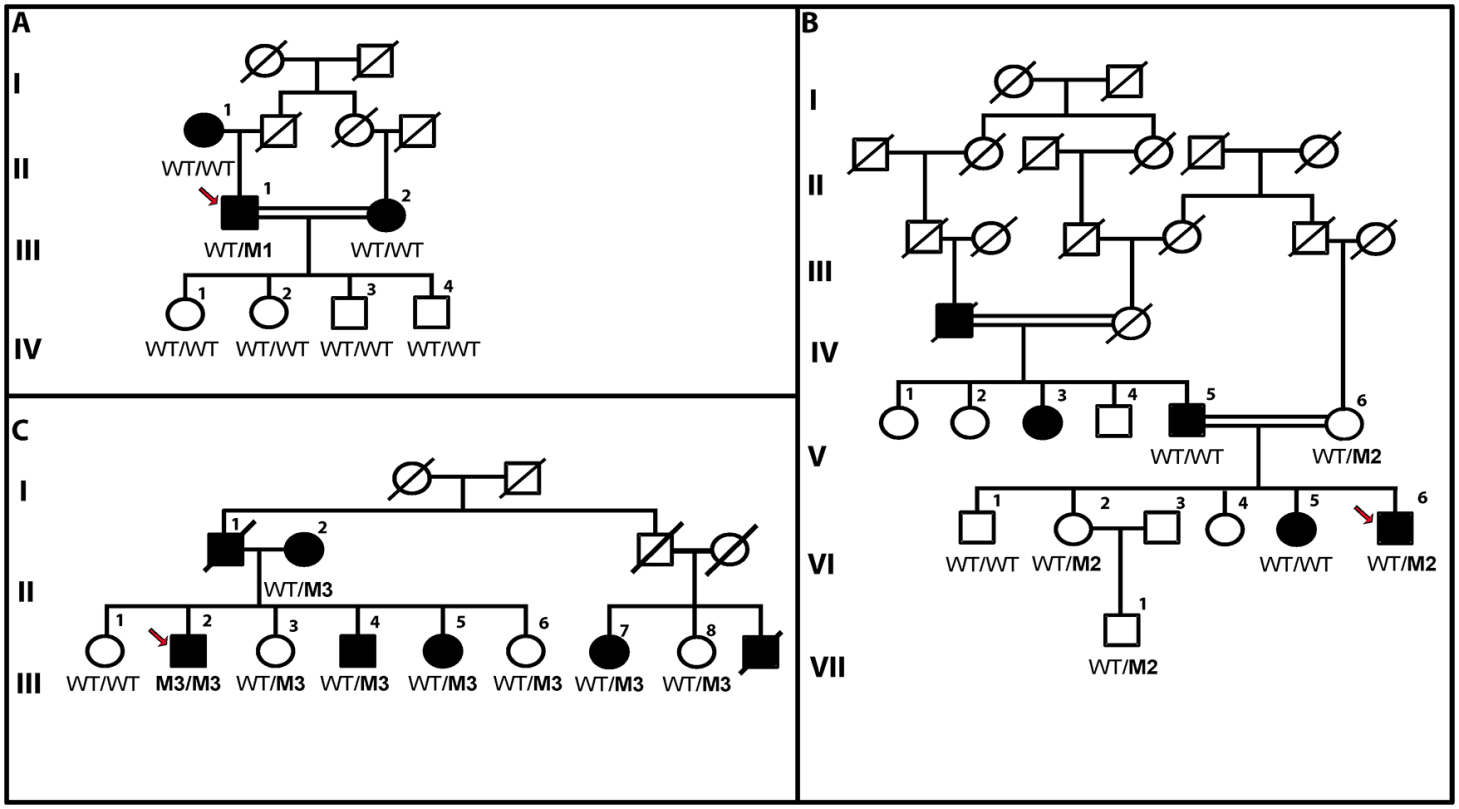
Pedigrees of consanguineous Pakistani glaucoma families, with a probable autosomal dominant inheritance pattern. Segregation analysis of three nonsynonmous variants: (**1A)** represents segregation of p.Arg49Cys, **(1B)** segregation of p.Arg237Gly change, **(1C)** shows p.Arg453Cys variant segregation, Variant allele is indicated with an “M1(p.Arg49Cys), M2(p.Arg237Gly) and M3(p.Arg453Cys)”, and the wildtype allele indicated with “WT” for the three variations. This demonstrates that the variants do not segregate with the disease in the respective families. The proband is indicated with an arrow.

Of these variants, three nonsynonymous variants (p.Arg49Cys, p.Arg237Gly, p.Arg453Cys) were not detected in control individuals (Table 1). However, none of the variants segregate with the disease in the family members of the probands (Fig 1). However individual III:2, who is homozygous for the pArg453Cys variant, has a more severe phenotype compared to his sibs. He was diagnosed with bilateral glaucoma in the age of 15 years. He was subjected to two trabeculectomies to control the IOP of both eyes (right 42mmHg; left 38mmHg). The cup-to-disc ratios of both eyes were also high (0.9). He lost the vision in both eyes at age 35, presenting visual acuities of 20/25 and 20/60 with tubular visual fields (less than 10°). He still needs to use prostaglandin analogs to lower his IOP.

Sequence analysis of the *ASB10* gene was further extended to a Pakistani cohort of 208 sporadic POAG patients and 151 controls. In addition to the above mentioned variants, 14 additional variants were identified in sporadic POAG patients. Of these, seven variants were nonsynonymous, 4 synonymous and 3 were intronic variants (Tables 1 and 2). Five nonsynonymous variants (p.Val62Met, p.Gln295His, p.Arg296Gln, p.Arg304Cys, p.Arg447His) identified in POAG patients were not detected in control individuals. Conversely, no nonsynonymous, synonymous and intronic variants were identified that are present in control individuals but not in POAG patients.

Association of each individual nonsynonymous, synonymous and intronic variant with POAG was assessed (Tables 1 and 2). One rare nonsynonymous variant (p.Arg453Cys) was detected in 6 sporadic POAG patients, but not in control individuals (p [uncorrected] = 0.04). However, this difference also did not remain significant after correction for multiple testing (p [corrected] = 0.62). One common synonymous variant (p.Ala290Ala) was detected heterozygously more frequently in POAG patients (49%) and probands (50%) compared to control individuals (33%), and the difference in genotype frequencies remained significant after correction for multiple testing (p [uncorrected] = 0.002, p [corrected] = 0.047).

One variant (c.1218+42T>C) was found more frequently in control individuals than in POAG patients but the frequencies of three genotypes in patients compared to controls shows a trend towards association (p [uncorrected] = 0.04), suggesting it might have a protective role, but this did not remain significant after correction for multiple testing (p [corrected] = 0.62).

Collectively, nonsynonymous variants were detected in 106/1468 (7.2%) patients, and in 18/694 (2.6%) controls after excluding the common variants (c.1075 G>A, c.1114 C>T, c.1204C>A). A significant difference in number of variants in patients compared to controls was observed (p-value 0.000006) upon combing the number of patients and controls of the current and previous two studies (Table 3).

**Table 3:** Burden test for rare nonsynonymous variants identified in POAG patients and controls in different cohorts.

	Pakistan	Iowa [19]	Germany and US [18]	Combined form 3 studies
Patient	23/238 (9.7%)	13/158 (8.2%)	70/1172 (6.0%)	106/1468 (7.2%)
Control	2/151 (1.3%)	3/82 (3.7%)	13/461 (2.8%)	18/694 (2.6%)
p-value	0.0005	0.27	0.008	0.000006

**Fig 2:**
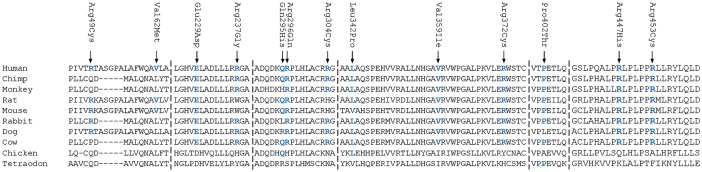
Amino acid conservation of amino acids of ASB10 in different species. Mutated amino acids conserved in humans and other species are shown in blue color.

Six of the 10 rare, nonsynonymous variants are predicted to be pathogenic by at least 2 pathogenicity prediction tools (p.Arg49Cys, p.Val62Met, p.Arg237Gly, p.Arg304Cys, p.Leu342Pro, p.Arg447His) (Table 1). In addition, seven variants (p.Glu229Asp, p.Arg296Gln, p.Val359Ile, p.Arg372Cys, p.Pro420Thr, p.Arg447His, p.Arg453Cys) affect amino acids that are highly conserved during evolution (Fig 2).

## Discussion

In a recent study, *ASB10* was identified as a novel gene for glaucoma at the GLC1F locus. A synonymous variant (c.765C>T; p.Thr255Thr) was identified in a large family with POAG, which was found to segregate with the disease. Further support was provided by the identification of *ASB10* variants in POAG patients from Oregon and Germany [17]. A few additional variants were identified in POAG patients from Iowa, but they were reported not to be significantly associated with POAG [18]. In the current study 10 rare amino acid changes were identified in 23/238 (9.7%) patients (Table 1) compared to 2/151 (1.3%) of the population matched controls (p-value 0.0005). Similarly in the study performed by Pasutto *et al* 70 patients were identified with 26 (6.0%) rare amino acid changes compared to 9 amino acid changes in 13 controls (2.8%) (p-value 0.008). However, in the Iowa cohort 11 rare amino acid substitutions were detected in 13/158 (8.2%) patients compared to 3/82 (3.7%) controls, and the difference was not statistically significant (p-value 0.27). Taken together, the burden of rare amino acid changes among all three cohorts, a significant association with POAG (p-value 0.000006) was observed.

The synonymous variant p.Thr255Thr identified by Pasutto *et al*. [17] was not found in a subsequent study from Iowa nor in the current study of Pakistani POAG patients. This suggests that Thr255Thr is a rare variant that may be population- or region-specific. The Thr255Thr was reported to affect an exonic splice enhancer, leading to aberrant splicing of the *ASB10* transcript.

In the current study we detected a significant association of another synonymous variant (c.870G>C; p.Ala290Ala), which was also reported previously in POAG patients from Germany, Oregon and Iowa. In the Iowa cohort the p.Ala290Ala variant (labeled as p.Ala275Ala) was also present at a higher frequency in POAG patients compared to controls with a marginally significant p-value 0.05 [18]. In Pakistani POAG patients a similar frequency of the heterozygous genotype (49%) was observed, and the frequency in controls (33%) was slightly lower than in the Iowa cohort (p-value 0.01; OR 1.76 [95% CI 1.10–2.83]). However, in the German cohort the frequency of the heterozygous genotype was higher in controls (57%) compared to POAG patients (47%) [17]. Therefore, the significance of this association in the pathogenicity of POAG is unclear.

Interestingly, both synonymous variants, p.Thr255Thr [17] and p.Ala290Ala, are located in one of the ankyrin repeats. The ankyrin repeat structure and number is an important determinant of the target substrate to which ASB10 protein is bound. Disruption of the repeat structure by altered splicing may thus affect the substrate binding of ASB10 [23]. Based on an *in silico* analysis of exonic splice enhancer sites, we assume that the effect of the p.Ala290Ala variant may be subtle as the affinity for SRp55 is predicted to be reduced by approximately 40%, while the splice enhancer (SF2/ASF) site affected by the p.Thr255Thr variant was completely lost. In a survey by Chen *et al* it has been observed that both nonsynonymous and synonymous polymorphisms have an equal probability of their association with the disease (1.46% versus 1.26% respectively) [24].

Three rare nonsynonymous variants were analyzed in family members, and did not segregate with the disease in these families. The ASB10 protein is expressed in the retina, the retinal ganglion cells, the trabecular meshwork, and the ciliary body. The ciliary body is involved in the production of aqueous humor, and the trabecular meshwork plays a phagocytic role to clear and regulate the outflow of aqueous humor. In the study of Pasutto *et al*, silencing of the *ASB10* gene was shown to lead to a higher resistance in the outflow of aqueous humor in a perfused segment organ culture [17]. Recently, evidence has also been provided for the involvement of ASB10 in the ubiquitin-mediated degradation pathways in trabecular meshwork cells [23].

In summary, the current study demonstrates that variants in *ASB10* are significantly associated with POAG in the Pakistani population. The current case-control study had 84% power to confirm the association. This study replicates the previous findings that sequence variants in the *ASB10* gene may act as a risk factor for glaucoma. Further studies in other populations are required to better understand the role of *ASB10* gene variants in glaucoma.
